# Next-Generation Sequencing for Evaluating the Soil Nematode Diversity and Its Role in Composting Processes

**DOI:** 10.3390/ijms242115749

**Published:** 2023-10-30

**Authors:** Anita Zapałowska, Andrzej Skwiercz, Anna Tereba, Czesław Puchalski, Tadeusz Malewski

**Affiliations:** 1 Department of Agriculture and Waste Management, Collegium of Natural Sciences, University of Rzeszów, St. Ćwiklinskiej 1a, 35-601 Rzeszów, Poland; 2 National Institute of Horticultural Research, Konstytucji 3 Maja 1/3, 96-100 Skierniewice, Poland; andrzej.skwiercz@inhort.pl; 3 Department of Forest Ecology, Forest Research Institute, Braci Leśnej 3, Sękocin Stary, 05-090 Raszyn, Poland; a.tereba@ibles.waw.pl; 4 Department of Bioenergetics, Food Analysis and Microbiology, Institute of Food Technology and Nutrition, Collegium of Natural Sciences, University of Rzeszów, St. Ćwiklińskiej 2D, 35-601 Rzeszów, Poland; cpuchlaski@ur.edu.pl; 5 Department of Molecular and Biometric Techniques, Museum and Institute of Zoology, Polish Academy of Sciences, 00-679 Warsaw, Poland; tmalewski@miiz.waw.pl

**Keywords:** dominant species, organic matter, environmental indicator, compost stability, molecular approaches

## Abstract

Biodiversity within composting systems involves a variety of microorganisms including nematodes. In the research, nematode populations were monitored within six simultaneously operating composting processes. These processes involved varying proportions of feedstock materials. The primary objective was to evaluate the consistency of nematode community succession patterns across the composting processes over a time of 3 months. During the study, samples were taken every month to isolate nematodes, determine the population density of the five trophic groups (per genus) and determine the dominant nematode species. It was shown that the bacterial-feeding community maintained dominance, while the fungus-feeding nematodes gradually increased in dominance as the maturation process progressed. The presence of predatory nematodes *Mononchoides* which were initially absent, along with the total absence of parasitic nematodes in the late stages of waste stabilization, serves as strong evidence for the reliable evaluation of the biodegradable waste processing level. Based on the obtained results, it is evident that the succession of nematode communities holds promise as a reliable method for evaluating compost maturity.

## 1. Introduction

Incorporating organic waste materials into agricultural forms is a part of the circular economy concept. It closes the loop by using waste materials as inputs for agricultural production, promoting resource efficiency and reducing waste generation. Organic waste materials are rich in essential nutrients like nitrogen, phosphorus, and potassium, as well as micronutrients. When these organic materials are recycled as fertilizers, they can help replenish soil nutrients, which are crucial for plant growth and crop production.

Recycling of organic waste products as fertilizers for agricultural fields has received big attention recently. There has been a significant surge in interest surrounding the practice of repurposing organic waste materials into fertilizers for agricultural purposes. This entails diverting organic waste away from residual waste streams and repurposing it as valuable fertilizer for use in agricultural fields. Understanding the biodiversity of composting systems, including nematode communities, can have implications for the management of composting operations, including optimization for agricultural use and minimizing potential environmental risks. Knowledge of nematode communities can inform composting practices to optimize the process. By monitoring nematode diversity and populations, compost operators can make adjustments to temperature, moisture, and aeration levels to ensure efficient decomposition and the production of high-quality compost. To be deemed advantageous for soil health and to confer associated benefits such as enhanced soil nutrient retention and disease suppression, the end product, known as compost, must meet stringent criteria. This includes being stable, fully matured, and devoid of any potential health or environmental hazards [1,2,3].

Nematodes are an essential, ubiquitous and abundant group of animals in compost. They are a diverse group of organisms that contain bacterial and fungal feeders, plant parasites, omnivores and predators [4]. Shifts in the nematode species composition, life strategies and feeding behavior during composting appear to be fairly consistent and, therefore, promising as a potential tool to assess compost maturity [5].

Different types of nematodes have distinct feeding habits, and their presence can indicate the decomposition status and nutrient content of the compost. An imbalance in nematode populations can suggest issues in the composting process.

The diversity and abundance of nematodes in compost can serve as an indicator of soil health and the efficiency of composting. The composition of nematode communities in compost can influence its effectiveness as a soil amendment. A balanced nematode community can contribute to improved soil quality and plant health when compost is applied to agricultural fields. Nematode community succession appears to be consistent and promising as a tool to assess compost maturity [5,6]

Identification of nematodes at the species level using specimen-based morphological identifications is difficult and time-consuming. Several molecular approaches have been used to supplement morphological methods and solve these problems with markable success [7,8,9]. Among them, next-generation sequencing (NGS) and metabarcoding have been gaining more attention for application to nematode community analyses [10,11,12].

The most widely used markers for nematoda metabarcoding are nuclear ribosomal RNA gene repeats (rDNA) [13,14,15]. Amplification of the D3 expansion segment of the 28S rDNA showed that it was able to detect ~90% of the sampled species [13].

In this research, we observed the nematode populations in six different composting processes. Each process involved different combinations of feedstock materials. The aim was to investigate whether the patterns of nematode community succession remained consistent across these composting processes as time progressed.

## 2. Results and Discussion

### 2.1. Compost Properties

The compost’s characteristics varied depending on its type. The nitrogen (N) content in the compost varied from 1.38% to 3.80% in the very beginning. Following the composting procedure, compost C3 exhibited the lowest nitrogen content at 1.01%, whereas compost C1 displayed the highest nitrogen content at 4.02% (Table 1). Among the various variants analyzed, compost C3 displayed the highest carbon-to-nitrogen (C:N) ratio. Depending on the composting duration, the average C:N ratio ranged from 38.55% at the beginning of the process to 37.72% at the end of the process, peaking on the 60th day of composting (41.11). Conversely, compost C1 showed the lowest carbon-nitrogen (C:N) content of 19.25% at the end of the process. Compost C3 also had the lowest average phosphorus (P_2_O_5_) content. Depending on the composting duration, the average P_2_O_5_ content ranged from 1.72 to 2.06 g kg^−1^. On the contrary, compost C1 had the highest P_2_O_5_ content, averaging between 10.82 and 13.00 g kg^−1^.

Regarding potassium (K_2_O) content, compost C2, C5, and C6 had the highest levels after the composting process (6.88 g kg^−1^, 7.63 g kg^−1^ and 7.28 g kg^−1^, respectively) (Table 1).

In the range of analyzed variants, after completion of the composting process, compost C1 displayed the highest concentration of lead (Pb), while compost C3 and C4 showed the highest concentration of chromium (Cr). Furthermore, among all the variants, compost C6 registered the highest average nickel (Ni) content, as shown in Table 2.

### 2.2. Nematode Succession

**Figure 1:**
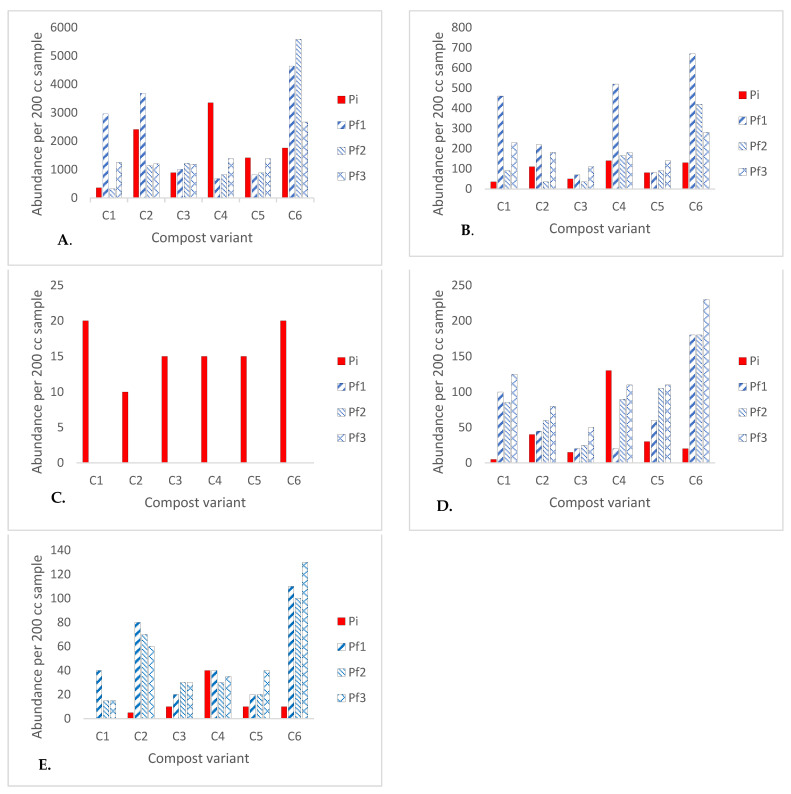
The nematode density per 200 cc compost sample at the level of five trophic groups: Bacterivores (**A**); Fungivores (**B**); Plant Parasitic Nematodes (PPN) (**C**); Omnivores (**D**); Predators (**E**) during the fourth data collection period (Pi- on the initial day of composting, Pf1- on the 30th day of composting, Pf2- on the 60th day of composting, Pf3- on the 90th day of composting).

**Figure 2:**
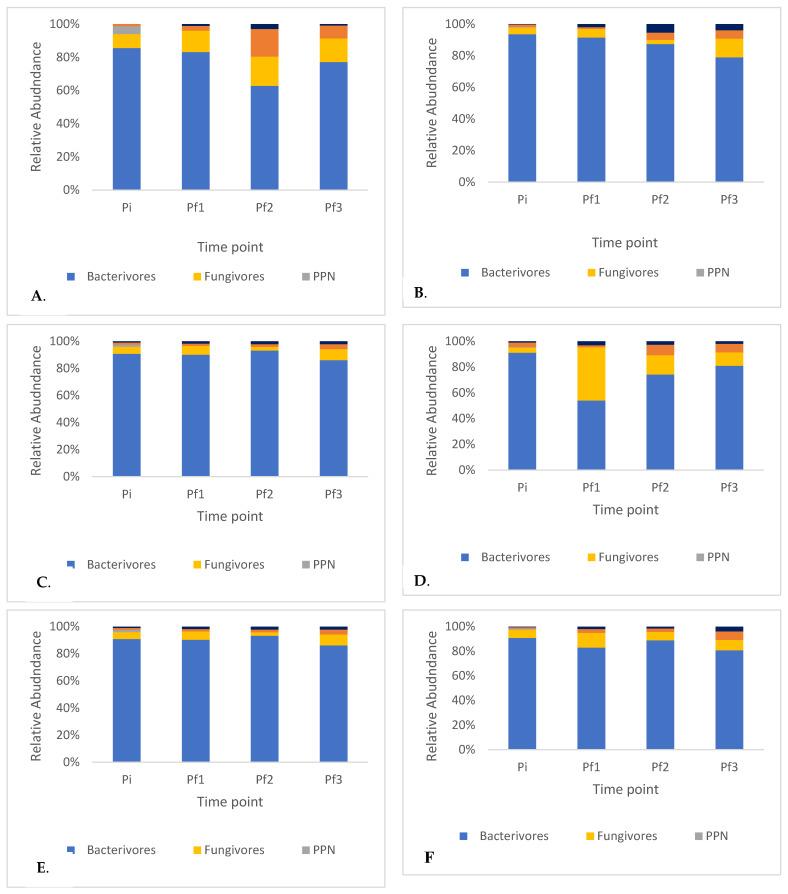
Relative abundance using morphological approach at the level of five trophic groups in six compost variants: compost C1 (**A**), compost C2 (**B**), compost C3 (**C**), compost C4 (**D**), compost C5 (**E**), compost C6 (**F**) during the fourth data collection period (Pi- on the initial day of composting, Pf1- on the 30th day of composting, Pf2- on the 60th day of composting, Pf3- on the 90th day of composting).

The fluctuations in population density among five different trophic groups of soil nematodes, observed in the studied samples, are depicted in correlation with the sequential stages of compost maturation. This alignment is based on a widely accepted framework comprising three successive phases, as supported by the consensus among most researchers [16,17]. The thermophilic stage is characterized by a rapid elevation in substrate temperature, particularly evident in mixtures that include grasses. In our research, the thermophilic phase spanned from the inception of the experiment until the fourth week. This was marked by a swift surge in the population density of bacterivorous nematodes (Figure 1A), which exhibit a brief individual development period, while the population of parasitic nematodes (Figure 1C) dwindled. Their presence ceased to be detected just one week into the thermophilic phase because temperatures during this composting phase exceeded 40 °C, a lethal threshold for plant parasitic nematodes (PPN) [18,19,20,21,22]. In the experiment, the compost cooling phase in the containers commenced during the fourth week from the initiation of the process. During this phase, the population densities of fungivorous nematodes (Figure 1B) started to rise, leading to a shift in the ratio between bacterivores and fungivores in favor of the latter. Furthermore, there was an increase in the populations of omnivorous and predatory nematodes, a phenomenon likely attributable to the greater abundance of food resources available (Figure 1D,E). During the next compost maturation phase, we observed subtle shifts in the densities of bacterivorous and fungivorous populations (Figure 2), which exhibit a robust increase in the initial two phases. The outcomes of our experiment align with the findings reported by Steel et al. [17]. Notably, the population densities of fungivorous nematodes closely resembled those of bacterivores. While there were slight disparities compared to Steel et al. [17] these variances could be attributed to the specific environmental conditions within our container setup. In both research papers, a significant emphasis was placed on analyzing nematode populations, particularly the bacterivorous ones, as key indicators of compost maturity. In essence, our experiment’s results affirm the conclusions drawn by these researchers, underscoring the efficacy of nematode population analysis as a reliable method for assessing compost maturity. Notably, the inclusion of E. fetida in one of our combinations expedited the proliferation of bacterivores and fungivores. This alteration shifted the proportion of population bacterivores to fungivores in favor of fungivores, deviating from the patterns observed in the aforementioned works.

To identify the genera and species of adult and immature nematodes, metabarcoding analysis was performed. Obtained sequence reads were filtered and low-quality reads were discarded. High-quality reads were checked for chimera, and the chimera was also removed. There were obtained between 1027 and 96,271 reads per sample. The high-quality chimera-free reads have been assigned to OTUs. There were detected nine nematode families: Aphelenchidae, Aphelenchoididae, Cephalobidae, Diplogastridae, Neodiplogastridae, Plectidae and Rhabditidae.

In the family Aphelenchoididae, reads were assigned to three genera Aphelenchoides, *Bursaphelenchus* and *Ektaphelenchus* but only in *Bursaphelenchus* reads were assigned to species level—*B. fungivorus*. Cephalobidae family consisted of two genera: *Cephalobus* and *Eucephalobus*, the last one represented by *E. striatus*. In the Diplogastridae family reads were assigned to family (Diplogastridae), genus (*Acrostichus and Demaniella*) or species (*Acrostichus floridensis*, *Acrostichus nudicapitatus*) level.

**Figure 3:**
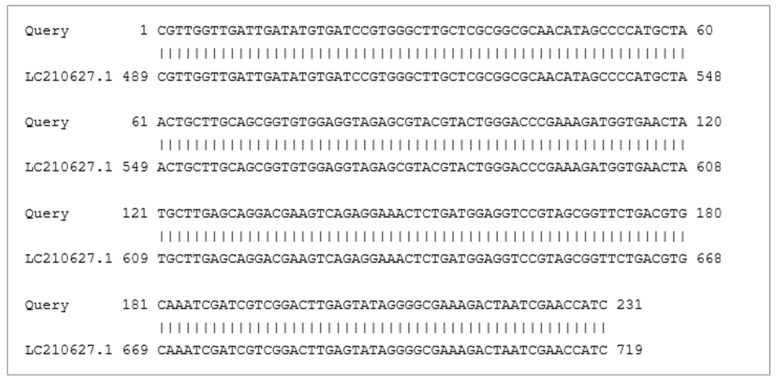
An example of comparison of sequence assigned to *A. floridensis* to a partial sequence of 28S ribosomal RNA (Acc no. LC210627.1).

BLASTn analysis of randomly chosen 100 OTU sequences assigned to *A. floridensis* and *A. nudicapitatus* showed that most of them have 100% identity to both reference sequences from the GenBank database. Figure 3 presents a sample comparison between the sequence attributed to *A. floridensis* and a partial 28S ribosomal RNA sequence (Acc no. LC210627.1).

In Neodiplogastridae reads were assigned to *Mononchoides* genus. In the Panagrolaimidae family *Panagrellus redivivus*, *Halicephalobus gingivalis and Halicephalobus* cf. *gingivalis* species were detected. There were detected two species (*Plectus acuminatus*, *Plectus cirratus*) from the Plectidae family. Some of the reads were assigned to the Rhabditidae family, nematoda environmental sample and other nematoda.

Most of the detected species or genera were low-abundance. The abundance of only seven species (*Acrostichus floridensis, Acrostichus nudicapitatus*, *Halicephalobus gingivalis*, *Halicephalobus* cf. *gingivalis*, *Oscheius onirici* and *Panagrellus redivivus*) and three OTUs identified to genus level (*Acrostichus*, *Mononchoides* and *Ektaphelenchus*) exceed 1% in at least one of analyzed samples (Table 3).

Species composition and abundance depended on the type of compost used and the composting time. In compost prepared from sewage sludge + sawdust (C1), the most abundant were *Acrostichus* (60.3%) followed by *Halicephalobus* cf. *gingivalis* (30.3%) and *Halicephalobus gingivalis* (8.0%). After 1 month of composting, their abundance decreased to 43.7%, 9.0% and 2.0%, respectively. After 2 months of composting, the abundance of *Acrostichus* stayed at approximately the same level (47%) while *H.* cf. *gingivalis* and *H. gingivalis* were not detected in analysed samples. These changes were associated with the appearance of *Mononchoides* (32.3% and 20.1% after 1 and 2 months, respectively) and after two months, OTUs were identified at the family level (Diplogastridae, 29.0%).

In compost prepared from biodegradable garden and park waste + sawdust (C3) *H.* cf. *gingivalis* and *H. gingivalis* were about two-fold more abundant (61.3% and 16.7%, respectively) than in compost prepared from sewage sludge (C1). Reads identified as *Acrostichus sp*. genus were only 2.0% but were found to be *A. floridensis* (17.7%) not detected in sewage sludge compost. Diplogastridae appeared early, after one month of composting.

In compost prepared from mixed sewage sludge and biodegradable garden and park waste (C3) abundance and changes during composting for Diplogastridae, *Acrostichus sp*., *H.* cf. *gingivalis* and *H. gingivalis* were similar to those in sewage sludge, but *A. floridensis* similar to biodegradable waste. Unlike the previous types, this type of compost had a low abundance of *Mononchoides*.

The addition of *E. fetida* to sewage sludge compost leads to the appearance of Diplogastridae (28.0%) and a two-fold decrease in *H. gingivalis* (from 8.0% to 3.7%) and *H.* cf. *gingivalis* abundances (from 30.3% to 15.0%). A high abundance of Diplogastridae remained also after one (21.7%) and two (36.1%) months of composting. *A. floridensis* was detected in one (2.3%) and two month (0.2%) compost.

Diplogastridae were also present in the garden and park waste compost but were two-fold (11.0%) less abundant than in sewage sludge. The opposite effect was found with the addition of *E. fetida* to garden and park waste compost on *A. floridensis* abundance. While in sewage sludge compost their amount ranged from 10.6 to 30.7% in the garden and park waste was 0.0–6.0%. In mixed compost Diplogastridae and *Halicephalobus* abundance were similar to sewage sludge.

Two species of nematoda were the most abundant: *Acrostichus* reaching 73.0% and *Halicephalobus* cf. *gingivalis* (61.3%). Our data are inline with previously published. *Acrostichus* have been reported from a wide range of habitats including fresh and polluted water, aquatic mulm and sewage [23]. *A. nudicapitatus* also is a widely distributed species reported from polluted water, dung as well as bark beetles [24]. *Halicephalobus* is a bacterial-feeding nematode that inhabits soil, soil-like environments and organic-rich substrates [5,25,26]. *H. gingivalis* is an opportunistic parasite of horses and other mammals causing halicephalobiasis [27,28,29].

Bacterial-feeding enrichment opportunists were most numerous during and directly after the heat peaks. Subsequently, the bacterial-feeding/predator community dominated and the fungal-feeding nematodes became more dominant during maturation, confirming general community patterns from previous experiments. Nematode abundances significantly fluctuated with temperature and the relative abundance of fungal-feeding nematodes increased as the duration of the curing process increased. The amount of fungal-feeding nematodes was associated significantly with both the duration of composting and temperature, and the F/(F + B) ratio was only significantly associated with the duration of composting. Steel et al. [5] should discuss the results and how they can be interpreted from the perspective of previous studies and of the working hypotheses. The findings and their implications should be discussed in the broadest context possible. Future research directions may also be highlighted.

The appearance of predatory nematodes (*Mononchoides*), which were absent at the beginning of the process, and the complete lack of parasitic nematodes in the final stage of waste stabilization confirm the effective assessment of the degree of processing of biodegradable waste. The research findings demonstrate that the levels of Pb, Cr, Cu, Ni, Cd, and Hg in the final composted material were considerably lower than the permissible standards set for sewage sludge used in agricultural applications [30,31]. The results from the nematological diagnosis align with the chemical analyses, both indicating that the produced material is suitable for agricultural use.

## 3. Materials and Methods

### 3.1. Study Site and Compost Preparation Procedure

The research took place in the Municipal Waste Landfill located at coordinates 50.22° N, 22.35° E, in the Podkarpackie (Subcarpathian) Province of Poland, spanning from September to December 2022. During this period, the mean air temperature for September was 15 °C, followed by 10.5 °C in October, 5.5 °C in November, and 1 °C in December.

Compost was prepared using different organic matter in various mass proportions (% by mass):

C1. Sewage sludge (80%) + Sawdust (20%);
C2. Sewage sludge (40%) + Sawdust (10%) + Biodegradable garden and park waste (50%);
C3. Biodegradable garden and park waste (90%) + Sawdust (10%);
C4. Sewage sludge (80%) + Sawdust (20%) + *Eisenia fetida;*
C5. Sewage sludge (40%) + Sawdust10%) + Biodegradable garden and park waste (50%) + *Eisenia fetida*;
C6. Biodegradable garden and park waste (90%) + Sawdust (10%) + *Eisenia fetida.*

Following the mixing process, the municipal waste blend was placed into six bioreactors, each of which was a thermally insulated plastic container with a cubic shape and a capacity of 1 m^3^. During the bioconversion process of substrates, it was necessary to employ mechanical mixing. The moisture content of the composting organic material was assessed on a weekly basis. Temperature readings provided confirmation of the presence of four distinct phases in the composting process, namely, the mesophilic, thermophilic, cooling, and maturation phases.

### 3.2. Chemical Analysis of Compost

Analyzes were performed according to the methodology described in the paper by Zapałowska et al. [32].

### 3.3. Morphological Identification of Nematodes

Samples were taken every month to isolate nematodes, determine the population density of the five trophic groups (per genus) and determine the dominant nematode species according to the methodology described in the paper by Zapałowska and Skwiercz [33].

### 3.4. DNA Extraction

For genetic analyses, nematodes were conserved in DESS [34]. For each sample, total DNA was extracted from the soil using a DNA Mini Kit (Syngen Biotech, Wrocław, Poland), following the manufacturer’s protocol except for a slight modification at the beginning of the procedure; 200 μL DLT1 buffer and 20 μL proteinase K solution were added to the nematode suspension, and the entire solution was vortexed and incubated over-night at 60 °C. Then 5 μL of proteinase K solution was added, vortexed and incubated for another 30 min. For each sample, DNA was eluted in 100 μL elution buffer. Purified DNA was stored at −20 °C. The DNA concentrations and purity of all samples were measured with a NanoDrop (Thermo Fisher Scientific, Waltham, MA, USA). The concentration of extracted DNA ranged from 11 ng/μL up to 100 ng/μL, and the 260/280 ratio was in the range of 1.86–2.07. The DNA concentration of all samples was normalized to 10 ng/μL for amplicon PCR.

### 3.5. Libraries Preparation

Multiplexed amplicon libraries were constructed according to the two-step PCR protocol described by Ahmed et al. 2019 [11]. This method consists of dual PCR amplification.

The first PCR uses amplicon-specific primers, including an Illumina adapter overhang (amplicon PCR), and the second, cycle-limited PCR is used for the incorporation of Illumina index adapters for multiplexing (index PCR). The D2-D3 segment of the 28S rDNA region was amplified using the primers Nex_D3FA and Nex_D3BR:

Nex_D3FA TCGTCGGCAGCGTCAGATGTGTATAAGAGACAGGACCCGTCTTGAAACACGGA

Nex_D3BR GTCTCGTGGGCTCGGAGATGTGTATAAGAGACAGCGAAGGAACCAGCTACTA

The primers Illumina sequencing adapters are given in italics above.

Briefly, amplicon PCR was conducted as described below. The PCR cocktail of 20 μL reaction volume comprised 10 μL KAPA HiFi Hot-Start ReadyMix (Roche, Basel, Switzerland), 1 μL of forward primer, and 1 μL of reverse primer at 5 Μm, 2 μL of nuclease-free water, and 2 μL of template DNA (20 ng). PCR reactions were carried out with the following program on a Veriti 96-Well Thermal Cycler (ThermoFisher Scientific, Waltham, MA, USA): initial denaturation for 3 min at 95 °C followed by 30 cycles of 30 s at 95 °C, 30 s at 55 °C, 30 s at 70 °C, and a final elongation cycle for 5 min at 72 °C. The first PCR product was purified with CleanNGS (CleanNA, Waddinxveen, The Netherlands). Following purification, 2 μL of the first PCR product was PCR amplified for final library construction containing the index using the NEBNext Multiplex Oligos for Illumina 96 Index Primers (New England Biolab, Ipswich, UK). The cycle condition for the second PCR was the following: initial denaturation for 3 min at 95 °C followed by 8 cycles of 30 s at 95 °C, 30 s at 55 °C, 30 s at 72 °C, and a final elongation cycle for 5 min at 72 °C. The second PCR product was purified the same way as the first PCR product. The resulting PCR products were pooled, and the final purified product was then quantified using qPCR according to the qPCR Quantification Protocol Guide (KAPA LibraryQuantification Kits for Illumina Sequencing platforms, Roche, Basel, Switzerland).

The paired-end (300 bp with V3 chemistry) sequencing was performed using the MiSeq platform (Illumina, San Diego, CA, USA).

### 3.6. Processing and Analysis of Sequencing Data

Reads quality was checked at FastQC [FastQC Project] and filtered using the Trimmomatic (version 0.38) [35] to exclude low-quality reads (Q < 20, sequences with any ambiguous (N) bases, more than six homopolymers). The chimera sequences identified by Mothur 1.31.2 [36] were discarded. To analyze community composition and assign taxonomic affiliations to the amplicon sequences, we used the software pipeline CCMetagen v1.2.3 (ConClave-based Metagenomics) [37] that utilizes the ConClave sorting scheme [38]. Taxonomic assignment was carried out utilizing the entire NCBI nucleotide collection. The criteria used for taxonomic assignment in CCMetagen were as follows: species-level similarity threshold of 98.41%, genus-level of 96.31%, family-level of 88.51%, order-level of 81.21%, class-level of 80.91%, and phylum-level of 50% [39].

## 4. Conclusions

Nematodes, recognized as vital members of the soil biota, have established themselves as key indicators of soil health. They provide important information on the dynamics of soil food webs. This research emphasizes the significance of nematode community succession as a reliable indicator for evaluating compost maturity. As it has been demonstrated, metabarcoding can be an efficient method for ecological studies such as this one.

## Figures and Tables

**Table 1:** Effect of compost variant on macroelements content during compost process (mean ± SD).

Content	Compost Variant	Composting Duration (Days)			
		0	30	60	90
N (%)	C1	3.80 ± 0.09	3.2 ± 0.08	2.96 ± 0.23	4.02 ± 0.32
	C2	3.72 ± 0.08	1.67 ± 0.05	2.77 ± 0.22	1.82 ± 0.14
	C3	1.38 ± 0.04	1.32 ± 0.10	1.11 ± 0.08	1.01 ± 0.08
	C4	3.5 ± 0.07	3.13 ± 0.25	3.01 ± 0.24	2.59 ± 0.20
	C5	3.54 ± 0.07	2.46 ± 0.19	2.34 ± 0.18	1.86 ± 0.14
	C6	1.53 ± 0.03	1.47 ± 0.11	2.07 ± 0.16	1.56 ± 0.12
P_2_O_5_ (g·kg^−1^ s.m.)	C1	12.00 ± 0.96	10.82 ± 0.86	12.61 ± 1.00	13.00 ± 1.04
	C2	5.81 ± 0.46	8.33 ± 0.66	6.80 ± 0.54	1.09 ± 0.01
	C3	1.93 ± 0.15	1.72 ± 0.13	2.06 ± 0.16	2.01 ± 0.16
	C4	9.27 ± 0.74	8.88 ± 0.71	10.06 ± 0.80	9.42 ± 0.75
	C5	10.22 ± 0.81	6.87 ± 0.53	7.58 ± 0.60	8.89 ± 0.71
	C6	2.38 ± 0.19	2.76 ± 0.22	5,98 ± 0.47	3.64 ± 0.29
K_2_O_5_ (g·kg^−1^ s.m.)	C1	4.11 ± 0.39	5.23 ± 0.49	6.00 ± 0.49	4.93 ± 0.30
	C2	1.00 ± 0.09	10.08 ± 0.99	8.40 ± 0.69	6.88 ± 0.55
	C3	11.76 ± 1.63	10.02 ± 0.97	5.82 ± 0.34	4.63 ± 0.37
	C4	3.66 ± 0.25	4.91 ± 0.48	6.06 ± 0.50	5.09 ± 0.40
	C5	4.10 ± 0.39	8.58 ± 0.81	9.18 ± 0.76	7.63 ± 0.61
	C6	5.36 ± 0.49	10.08 ±0.10	10.62 ± 1.09	7.28 ± 0.58
C:N (%)	C1	19.81 ± 0.19	24.19 ± 0.30	26.14 ± 0.26	19.25 ± 0.91
	C2	12.79 ± 0.23	25.74 ± 0.73	12.74 ± 0.21	23.57 ± 0.86
	C3	38.55 ± 0.15	35.07 ± 0.20	41.44 ± 0.19	37.72 ± 0.54
	C4	22.8 ± 0.28	23.06 ±0.75	23.68 ± 0.27	28.06 ± 0.42
	C5	15.16 ± 0.17	17.64 ± 0.18	21.02 ± 0.26	21.29 ± 0.38
	C6	30.45 ± 0.34	32.38 ±0.27	23.91 ± 0.21	30.32 ± 0.19

**Table 2:** Effect of compost variant on microelements content during compost process (mean ± SD).

Total Metal Concentration	Compost Variant	Composting Duration (Days)			
		0	30	60	90
Pb (mg∙kg^−1^ s.m)	C1	11.0 ± 0.88	15.0 ± 1.2	8.5 ± 0.68	10.0 ± 0.8
	C2	10.0 ± 0.80	<8.0	<8.0	<8.0
	C3	<8.0	9.8 ± 0.78	<8.0	<8.0
	C4	<8.0	<8.0	<8.0	<8.0
	C5	8.20 ± 0.65	<8.0	<8.0	8.0
	C6	6.0 ± 0.48	<8.0	<8.0	<8.0
Chr (mg∙kg^−1^ s.m)	C1	<10	<10	<10	<10
	C2	<10	<10	<10	<10
	C3	<10	<10	<10	12.0 ± 0.96
	C4	11.0 ± 0.91	<10	<10	12.0 ± 0.93
	C5	11.0 ± 0.89	<10	<10	11.0 ± 0.90
	C6	16.0 ± 1.28	12.0 ± 0.96	13.0 ± 1.04	23.0 ± 1.84
Cu (mg∙kg^−1^ s.m)	C1	77.0 ± 6.14	65.0 ± 5.2	55.0 ± 4.01	70.0 ± 5.6
	C2	31.0 ± 2.48	13.0 ± 1.04	39.0 ± 3.12	58.0 ± 4.64
	C3	6.90 ± 0.52	9.2 ± 0.76	5.2 ± 0.41	16.0 ± 1.28
	C4	88.0 ± 7.01	67.0 ± 5.36	81.0 ± 6.48	84.0 ± 6.72
	C5	78 ± 6.01	55.0 ± 4,39	42.0 ± 3.36	45.0 ± 3.6
	C6	9.2 ± 0.73	11.0 ± 0.88	14.0 ± 1.12	16.0 ± 1.28
Ni (mg∙kg^−1^ s.m)	C1	5.7 ± 0.45	<5.0	<5.0	< 5.0
	C2	<5.0	<5.0	<5.0	< 5.0
	C3	<5.0	<5.0	<5.0	5.3 ± 0.42
	C4	6.3 ± 0.50	<5.0	5,7 ±	< 5.0
	C5	6.8 ± 0.54	6.4 ± 0.51	<5.0	5.1 ± 0.40
	C6	11.0 ± 0.88	7.1 ± 0.65	7.7 ± 0.61	11.0 ± 0.88
Cd (mg∙kg^−1^ s.m)	C1	0.473 ± 0.03	0.467 ± 0.03	0.311 ± 0.02	0,44 ± 0.03
	C2	0.30 ± 0.02	<0.3	0.32 ± 0.02	0.42 ± 0.03
	C3	<0.3	<0.3	<0.3	0.34 ± 0.03
	C4	0.568 ± 0.04	0.428 ± 0.03	0.392 ± 0.03	0.5 ± 0.05
	C5	0.456 ± 0.03	0.607 ± 0.04	<0.3	0.38 ± 0.03
	C6	<0,3	0.371 ± 0.02	<0.3	0.35 ± 0.03
Zn (mg∙kg^−1^ s.m)	C1	302 ± 24.1	225 ± 18.0	241 ± 18.9	253 ± 20,2
	C2	168 ± 13.4	54.0 ± 4.32	157 ± 12.1	223 ± 17.8
	C3	42.0 ± 3.06	46.0 ± 3.68	28.0 ± 2.24	91.0 ± 7.28
	C4	336 ± 25.9	288 ± 18.8	274 ± 21.9	274 ± 21.91
	C5	313 ± 24.9	248 ± 19.0	147 ± 11.7	232 ± 18.56
	C6	51.0 ± 4.08	57.0 ± 4.02	63 ± 5.25	78.0 ± 6.24
Hg (mg∙kg^−1^ s.m)	C1	0.243 ± 0.01	0.176 ± 0.01	0.133 ± 0.01	0.132 ± 0.01
	C2	0.057 ±0.01	0.039 ± 0.00	0.047 ± 0.00	0.104 ± 0.00
	C3	0.035 ±0.00	0.032 ± 0.00	0.034 ± 0.00	0.026 ± 0.00
	C4	0.134 ±0.01	0.168 ± 0.01	0.172 ± 0.01	0.18 ± 0.01
	C5	0.134 ± 0.01	0.074 ± 0.00	0.076 ± 0.00	0.066 ± 0.00
	C6	0.037 ± 0.00	0.041 ± 0.00	0.023 ± 0.00	0.042 ± 0.00

**Table 3:** Dominance of nematode (%) for various compost variants studied (mean ± SD). Only nematodes whose abundance in at least one sample is equal to or greater than 1% are listed in the table.

Species	Compost Variant	Composting Duration (Days)		
		30	60	90
Diplogastridae	C1	<0.1	<0.1	29.1 ± 5.23
	C2	<0.1	<0.1	27.7 ±± 4.03
	C3	<0.1	25.0 ± 3.19	23.2 ± 3.41
	C4	28.0 ± 4.21	21.7 ± 3.19	37.2 ± 4.14
	C5	28.0 ± 3.67	21.7 ± 4.15	36.1 ± 4.96
	C6	11.0 ± 3.11	4.3 ± 1.48	21.8 ± 4.13
*Acrostichus nudicapitatus*	C1	0.1 ± 0.04	<0.1	<0.1
	C2	<0.1	<0.1	<0.1
	C3	<0.1	<0.1	<0.1
	C4	<0.1	<0.1	0.1 ± 0.09
	C5	<0.1	<0.1	0.1 ± 0.08
	C6	<0.1	6.2 ± 1.42	<0.1
*Acrostichus sp*.	C1	59.2 ± 5.11	41.2 ± 9.16	46.9 ± 4.31
	C2	72.9 ± 2.13	40.6 ± 2.41	49.9 ±4.06
	C3	1.9 ± 0.47	40.1 ± 3.44	25.6 ± 3.24
	C4	49.8 ± 6.52	37.6 ± 4.16	54.8 ± 6.32
	C5	50.1 ± 7.32	37.9 ± 5.42	54.9 ± 7.38
	C6	10.7 ± 2.02	25.4 ± 4.62	27.9 ± 3.43
*Acrostichus floridensis*	C1	<0.1	<0.1	<0.1
	C2	4.3 ± 0.27	16.9 ± 1.55	10.3 ±0.21
	C3	16.9 ± 2.72	10.4 ± 2.09	30.5 ± 4.68
	C4	<0.1	2.2 ± 0.33	0.3 ± 0.11
	C5	<0.1	2.1 ± 0.41	0.2 ± 0.04
	C6	<0.1	5.9 ± 1.22	2.1 ± 0.61
*Mononchoides sp*.	C1	0.1 ± 0.02	32.3 ± 0.58	20.1 ± 3.81
	C2	<0.1	1.1 ± 0.21	<0.1
	C3	<0.1	1.1 ± 0.21	<0.1
	C4	0.5 ± 0.21	31.3 ± 3.17	1.9 ± 0.08
	C5	0.5 ± 0.16	31.3 ± 4.67	1.9 ± 0.33
	C6	1.3 ± 0.46	14.0 ± 2.61	2.0 ± 0.36
*Ektaphelenchus sp*.	C1	<0.1	<0.1	<0.1
	C2	<0.1	<0.1	<0.1
	C3	<0.1	1.3 ± 0.62	<0.1
	C4	<0.1	<0.1	0.1 ± 0.04
	C5	<0.1	<0.1	0.1 ± 0.05
	C6	<0.1	<0.1	<0.1
*Halicephalobus sp.*	C1	0.1 ± 0.06	<0.1	<0.1
	C2	<0.1	<0.1	<0.1
	C3	<0.1	<0.1	<0.1
	C4	<0.1	<0.1	<0.1
	C5	<0.1	<0.1	<0.1
	C6	0.3 ± 0.11	<0.1	6.9 ± 0.94
*Halicephalobus gingivalis*	C1	7.9 ± 1.35	1.9 ± 0.42	<0.1
	C2	3.9 ± 0.16	5.9 ± 1,64	<0.1
	C3	16.4 ± 3.91	1.2 ± 0.31	6.4 ± 1.16
	C4	3.5 ± 0.52	0.1 ± 0.07	<0.1
	C5	3.4 ± 1.22	0.1 ± 0.04	<0.1
	C6	16.8 ± 2.02	6.4 ± 1.41	<0.1
*Halicephalobus* cf. *gingivalis*	C1	30.3 ± 2.21	8.9 ± 1.06	<0.1
	C2	16.7 ± 1.42	23.5 ± 2.46	<0.1
	C3	60.9 ± 3.47	1.3 ± 0.43	1.9 ± 0.43
	C4	14.9 ± 2.11	1.2 ± 0.11	<0.1
	C5	15.0 ± 2.47	1.1 ± 0.32	<0.1
	C6	52.8 ± 7.43	24.1 ± 3.68	2.1 ± 0.56
*Oscheius onirici*	C1	<0.1	<0.1	<0.1
	C2	<0.1	<0.1	<0.1
	C3	<0.1	<0.1	<0.1
	C4	0.8 ± 0.21	<0.1	1.2 ± 0.70
	C5	0.9 ± 0.16	<0.1	0.9 ± 0.11
	C6	0.1 ± 0.04	<0.1	<0.1
*Panagrellus redivivus*	C1	<0.1	<0.1	<0.1
	C2	<0.1	0.2 ± 0.12	0.1 ± 0.04
	C3	<0.1	<0.1	0.2 ± 0.15
	C4	<0.1	<0.1	<0.1
	C5	<0.1	<0.1	<0.1
	C6	<0.1	1.0 ± 0.04	0.6 ± 0.04
Nematoda environmental sample	C1	0.1 ± 0.02	10.7 ± 2.51	1.3 ± 0.32
	C2	0.1 ± 0.12	8.1 ± 0.51	9.9 ± 2.49
	C3	0.3 ± 0.28	1.5 ± 0.32	<0.1
	C4	0.4 ± 0.16	2.9 ± 0.85	3.4 ± 0.79
	C5	0.3 ± 0.07	2.8 ± 0.81	3.2 ± 0.17
	C6	1.4 ± 0.11	1.3± 0.12	2.6 ± 0.15
Other nematoda	C1	1.2 ± 0.06	1.4 ± 0.49	2.3 ± 0.79
	C2	0.7 ± 0.19	2.6 ± 0.19	1.6 ±0.12
	C3	1.4 ± 0.72	1.3 ± 0.69	1.1 ± 0.17
	C4	1.3 ± 0.41	1.5 ±0.13	2.9 ± 0.39
	C5	1.5 ± 0.15	1.6 ± 0.48	3.1 ± 0.24
	C6	2.1 ± 0.16	1.7 ± 0.14	3.6 ± 0.12

## Data Availability

Not applicable.
